# Localization of Deep Brain Stimulation Contacts Using Corticospinal/Corticobulbar Tracts Stimulation

**DOI:** 10.3389/fneur.2017.00239

**Published:** 2017-05-31

**Authors:** Julien F. Bally, Maria-Isabel Vargas, Judit Horvath, Vanessa Fleury, Pierre Burkhard, Shahan Momjian, Pierre Pollak, Colette Boex

**Affiliations:** ^1^Department of Neurology, University Hospitals of Geneva (HUG), Geneva, Switzerland; ^2^Department of Neurology, Movement Disorders Research Center, Toronto Western Hospital, University of Toronto, Toronto, ON, Canada; ^3^Department of Neuroradiology, University Hospitals of Geneva (HUG), Geneva, Switzerland; ^4^Department Neurosurgery, University Hospitals of Geneva (HUG), Geneva, Switzerland

**Keywords:** DBS, current spread, PD, electromyography, MRI, corticospinal/corticobulbar tract

## Abstract

**Background:**

Successful deep brain stimulation (DBS) in Parkinson’s disease (PD) requires optimal electrode placement. One technique of intraoperative electrode testing is determination of stimulation thresholds inducing corticospinal/corticobulbar tracts (CSBT) motor contractions.

**Objective:**

This study aims to analyze correlations between DBS electrode distance to CSBT and contraction thresholds, with either visual or electromyography (EMG) detection, to establish an intraoperative tool devoted to ensure safe distance of the electrode to the CSBT.

**Methods:**

Twelve PD patients with subthalamic nucleus DBS participated. Thresholds of muscular contractions were assessed clinically and with EMG, for three different sets of stimulation parameters, all monopolar: 130 Hz high-frequency stimulation (HFS); 2 Hz low-frequency stimulation with either 60 or 210 µs (LFS-60, LFS-210). The anatomical distance of electrode contacts to CSBT was measured from fused CT-MRI.

**Results:**

The best linear correlation was found for thresholds of visually detected contractions with HFS (*r*^2^ = 0.63, *p* < 0.0001) when estimated stimulation currents rather than voltages were used. This correlation was found in agreement with an accepted model of electrical spatial extent of activation (*r*^2^ = 0.50). When using LFS, the correlation found remained lower than for HFS but increased when EMG was used. Indeed, the detection of contraction thresholds with EMG versus visual inspection did allow more frequent detection of face contractions, contributing to improve that correlation.

**Conclusion:**

The correlation between electrode distance to the CSBT and contraction thresholds was found better when estimated with currents rather than voltage, eliminating the variance due to electrode impedance. Using LFS did not improve the precision of that evaluation, but EMG did. This technique provides a prediction band to ensure minimum distance of the electrode contacts to the CSBT, integrating the variance that can be encountered between prediction of models and practice.

## Introduction

Deep brain stimulation (DBS) is an established procedure to treat motor symptoms of patients with Parkinson’s disease (PD). Current propagation toward the corticospinal/corticobulbar tracts (CSBT) can preclude further increase of the stimulation intensity and adequate patient care; as such it is important to ensure minimum distance from the DBS electrode to the CSBT (1, 2). The stimulation threshold of CSBT is clinically assessed intraoperatively by examining visually small muscle group contractions (2). Intraoperative stimulation has been used in brain tumor resections to prevent infarct of CSBT (3). Previously, the correlation between the distance of the resection site to CSBT and the stimulation amplitude had been studied, with different stimulation parameters than those in DBS procedures, through electromyography (EMG) and under general anesthesia (4–6).

In the DBS field, a linear correlation between the stimulation thresholds of CSBT side effects and the distance to CSBT has been found, allowing for the assessment of the position between CSBT to DBS electrodes (7). In this initial study, the stimulation amplitudes were described with voltages, and stimulation thresholds were determined visually. Aims of the present study are (i) to analyze the correlation between the distance of DBS contacts to CSBT and the amplitude of stimulation expressed with both voltage (volts) and estimated current (milliamperes), to establish an intraoperative tool devoted to ensure safe distance of the electrode to the CSBT; (ii) to determine if this correlation was stronger using low-frequency stimulation (LFS) or high-frequency stimulation (HFS) (i.e., 2 versus 130 Hz) and for pulse durations of 60 versus 210 µs, as used in centers intraoperatively; and (iii) to examine whether this correlation could be stronger using EMG beyond visually detected muscle contractions.

## Materials and Methods

### Patients

Thirteen patients agreed to participate in the study. Both medications and contralateral implanted pulse generator (IPG) were unchanged during the study, except for the patients who had an Activa PC (Medtronic, Minneapolis, MN, USA) for which it was not possible to maintain the contralateral subthalamic nucleus (STN) on HFS stimulation. One patient (P7) was excluded because of very high amplitude rest tremor. Twelve patients were included (6 female, 6 male, all right-handed; median age = 62.5 years; interquartile range: 58.5, 71.5 years; minimum: 51 years, maximum: 76 years). Duration of PD at surgery time was on average 12.3 years (standard deviation: 3.7 years).

Efficacy of STN-DBS was evaluated using the Movement Disorders Society-Unified Parkinson’s Disease Rating Scale (MDS-UPDRS) part III (motor part) in the OFF medication/ON stimulation condition 1 year after surgery, in reference to the OFF-medication condition evaluated before surgery. Mean improvement in MDS-UPDRS-III score was 48.3%, SD: 28.3, which is consistent with other studies (8–10) and a meta-analysis (11).

All patients were implanted under local anesthesia by the same neurosurgeon (SM) and received the bilateral quadripolar DBS lead 3389 (Medtronic Inc., Minneapolis, MN, USA) according to previously published methodology (12, 13).

This study was conducted according to ethical guidelines of the Declaration of Helsinki and was approved by the Ethical Committee of the University Hospitals of Geneva (ref. no. 14-230). All patients gave and signed an informed consent.

### Stimulation

Stimulation was applied through commercially available IPGs (Activa PC or SC; Medtronic, Minneapolis, MN, USA). Solely monopolar configuration was applied, one single contact of the electrode was stimulated in reference to the case of the pulse generators. While IPGs can deliver voltage- or current-controlled stimulation, this latter option is not available with the Medtronic Activa at frequencies lower than 30 Hz. Since we wanted to compare LFS versus HFS, the amplitudes of stimulation were delivered in volts, and impedances were measured at the end of each session, always with 1.5 V, for every contact in every patient. Applied currents were estimated with the Ohm’s law: current (mA) = voltage (V)/impedance [Ohms]. We verified with an externalized Activa-IPG that the voltage provided for HFS and LFS was of similar amplitude with 60 µs pulse duration. As previously described, IPGs provide first cathodic pulses of different amplitudes for different pulse widths [i.e., asymmetrical biphasic pulses, short duration cathodic first pulse, long duration anodic second pulse (14, 15)]. Hence, the voltage of the cathodic pulse was measured. Nevertheless, that voltage was found different from the one displayed by the IPG and is approximately 4% lower. To improve correlation precision, we included in the analysis this estimated—4% difference in voltage. With the same externalized Activa-IPG, we measured the difference in the amplitude (V) of the cathodic pulses for 60 and 210 µs and found an average difference of—1.5% (lower voltage with 210 µs, in consequence of electrical charge balancing). Therefore, we decreased by 1.5% the stimulation thresholds obtained with 210 µs before analyzing correlations. The applied currents were estimated with the Ohm’s law after the application of these corrections.

The session started with the chronic parameters, i.e., 130 Hz/60 μs. Patients were evaluated in their ON-medication state. The voltage was increased progressively until the first-evoked motor contraction was visually observed on the contralateral upper limb’s muscles and on the face. The stimulation threshold was defined as the voltage evoking that first contraction. The experiment was aborted when a side effect occurred before muscle contraction, e.g., dizziness, ill-being sensation, etc. This procedure was repeated for every four contacts of the right STN electrode. The same procedure was repeated for LFS, i.e., 2 Hz; contractions were visually and EMG detected.

### Clinical Detection of Contractions

Contractions were visually detected by coauthor JB, neurologist, focusing on upper and lower face muscles and distal contralateral hand muscles. This author was guiding the stimulation, therefore, was always aware if stimulation was ON or OFF as well as amplitude.

### Surface EMG

Surface electrode pairs (DSE 3115, Medtronic Xomed Inc., Jacksonville, FL, USA) were used to record EMG activity (bipolar recording). Monitored muscles were the contralateral thenar group and first interosseus/lumbrical muscles (upper limb) and orbicularis oculi and orbicularis oris (face).

**Figure 1:**
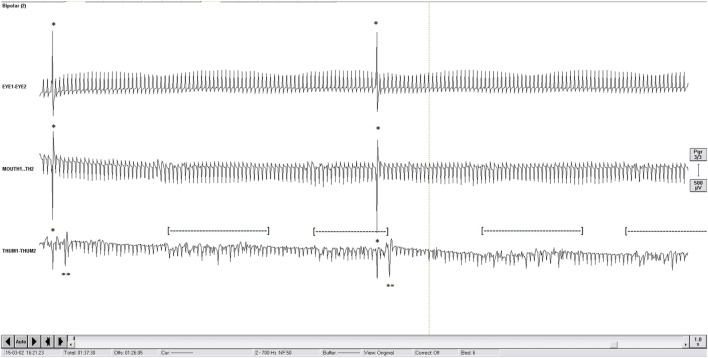
One second display of electromyography (EMG) recording (2 Hz). First channel, orbicularis oculi “EYE1-EYE2”; middle channel: orbicularis oris “MOUTH1-TH2”; bottom channel: thenar group “THUM1-THUM2.” To be noticed: continuous artifacts of contralateral high-frequency stimulation at 130 Hz; ipsilateral 2 Hz stimulation artifact (*); short latency EMG response of thenar group (**); 5 Hz Parkinsonian rest tremor recorded on the thenar group ([---]); of note, the short latency EMG response could be clearly seen even with the presence of the Parkinsonian tremor.

Electromyography was sampled at 5 kHz and band-passed filtered [(1; 700)Hz; BrainAmp MR plus, Brain Products Gmbh, Munich, Germany]. Ground electrode was a surface electrode located on the lateral aspect of the frontal bone, anterior to the temporal muscle. EMG activity was visually detected from EMG recordings by coauthor CB during the LFS sessions only, as HFS artifacts prevented the detection of EMG activity. This author was scrutinizing the EMG activity and was not aware of the intensity of stimulation. Brief muscular contractions were detected from EMG when they occurred shortly after the stimulation artifacts at latencies around 10 ms for the face and around 18 ms for the hand and at amplitudes higher than the 130 Hz artifacts from the contralateral not studied left electrode (Figure 1).

### Imaging and Determination of the Distance to the CSBT

Each patient had a detailed preoperative MRI (Siemens Trio 3.0 T scanner). The technical protocol included high resolution 3DT2, 3DFLAIR, axial T2*, axial FSET2, and 3DT1 sequences after administration of gadolinium. The 3DT2 sequence (TE = 223 ms, TR = 2,400 ms, field of view = 450 mm^2^, matrix = 448, slice thickness = 1.0 mm) was fused (16) with a postoperative CT-scan [Siemens Somatom Definition Flash (Siemens, Erlangen, Germany)] or a GE Discovery 750HD (GE Healthcare, Milwaukee, WI, USA).

The software used was the Integrated Registration, AW Volume Share 4.6, GE Healthcare. The program made a rigid-body registration using mutual registration with a two-pass-transform estimation (for rotation and translation). Image alignment was visually evaluated. In cases in which alignment was not satisfactory by use of the automatic alignment option, it was completed by use of the manual alignment option.

**Figure 2:**
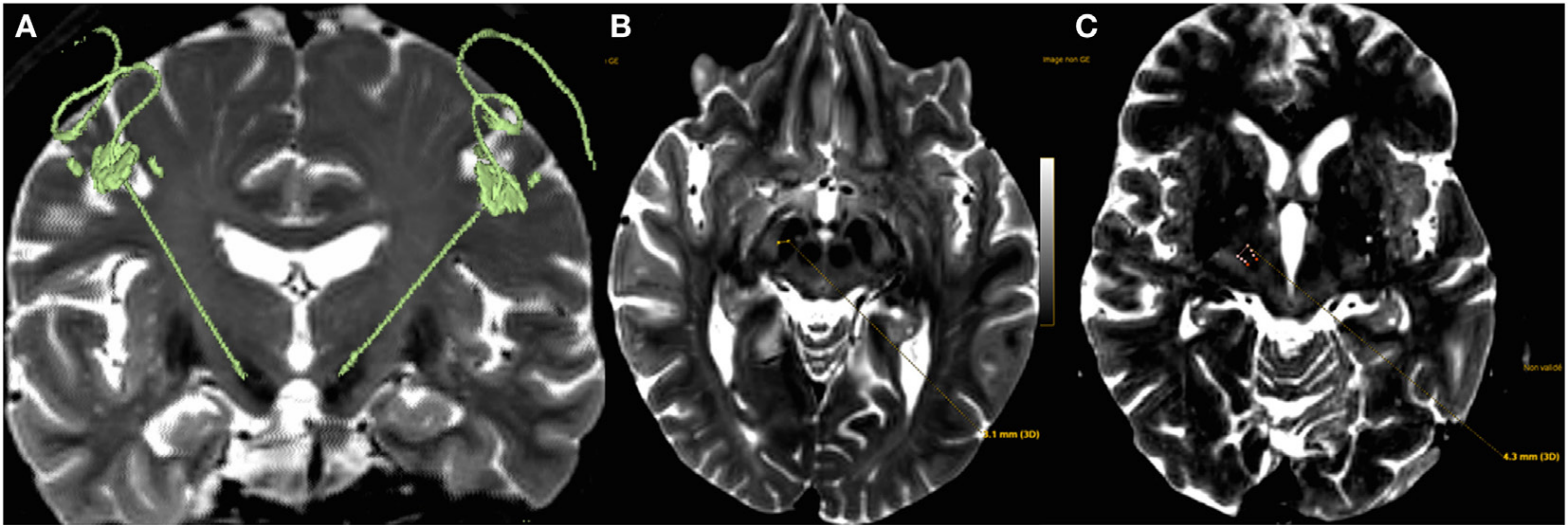
**(A)** 3D T2 sequence fused with postoperative CT showing electrodes placement within both subthalamic nucleus. **(B)** The first boundary of the “distance measurement tool” was adjusted on the center of the deep brain stimulation (DBS) contact visualized on the coronal plane of the CT image, here visible on an axial plane (T2-MRI; medial yellow point), extracted from the MRI-CT fusion; the lateral yellow point is the medial border of the corticospinal/corticobulbar tracts (CSBT) and was adjusted on the axial plane (MRI window). **(C)** For dorsal contacts (all four DBS contacts are represented medially, the most ventral one in red, the most dorsal one being the most anterior), the closest border of the CSBT to the electrode is no more medial but anterior or antero-medial.

The distance of each electrode contact’s center to the closest border of CSBT was measured: the first boundary of the “distance measurement tool” (DMT) was adjusted on the contact’s center visualized on the CT-coronal plane, extracted from the MRI-CT fusion; then the second boundary of the DMT was adjusted to get the smallest distance to the antero-medial border of CSBT visualized on the MRI-axial plane extracted from the MRI-CT fusion (Figure 2).

### Statistics and Fitting with Model of Electrical Spread Activation

All analyses were performed using SigmaStat 3.11 (Systat Software Inc., Richmond, CA, USA). Linear correlations between the distance of the electrode to CSBT and contraction thresholds were performed for HFS and LFS with computation of the Pearson’s correlation coefficients, once normality test (Kolmogorov–Smirnov test) and the test of equal variance for residuals were passed. In that case, 95% confidence bands of the regression and the 95% prediction bands were computed. In case normality test or equal variance test failed, the Spearman’s correlation coefficient was computed.

Pearson’s correlation coefficients were also computed to evaluate the goodness of fitting with the model of electrical spread of activation of Kuncel et al. (17). This model was also adapted for currents, dividing the voltage by the average impedance measured in our series of patients, i.e., 1,300 Ω.

Differences between proportions of face or hand responses were examined with *z*-tests (two-tailed test).

### Missing Values

We identified four types of missing values: the most frequent was abortion of the experiment when patients experienced a non-motor side-effect, a second type was when no contraction was observed even at the maximal amplitude, the third was if parkinsonian rest tremor prevented visual detection of contractions, and the fourth was when the patient was too tired to continue the experiment. Missing values were ignored.

## Results

Significant correlation was found between the distance of the DBS electrode to the CSBT and the stimulation threshold of contractions for HFS and LFS (Table 1).

**Table 1:** Coefficients of linear correlation (Spearman and Pearson’s correlation coefficients when adapted; Coef.) and significance of correlations, for each condition of measurements, showing the correlation between the corticospinal/corticobulbar tracts (CSBT) contraction thresholds and the deep brain stimulation electrode’s distance to the CSBT.

		Contractions visually detected		Contractions electromyography (EMG) detected	
		Coef.	*p*<	Coef.	*p*<
Stimulation parameters (threshold expressed in voltage, V)	High-frequency stimulation (HFS)				
	Spearman	0.54	0.005	NA	
	Low-frequency stimulation (LFS)-60				
	Spearman	0.45	0.01	0.62	0.005
	Pearson	NA	NA	0.36	0.005
	LFS-210				
	Spearman	0.50	0.005	0.53	0.005
	Pearson	0.33	0.005	0.44	0.0001

Stimulation parameters (threshold expressed in current, mA)	HFS				
	Spearman	0.72	0.0001	NA	
	Pearson	**0.63**	**0.0001**	
	LFS-60				
	Spearman	0.43	0.005	0.74	0.0005
	Pearson	NA	NA	NA	NA
	LFS-210				
	Spearman	0.61	0.0005	0.71	0.0001
	Pearson	NA	NA	NA	NA

*Electromyography is not feasible at HFS*.

**Figure 3:**
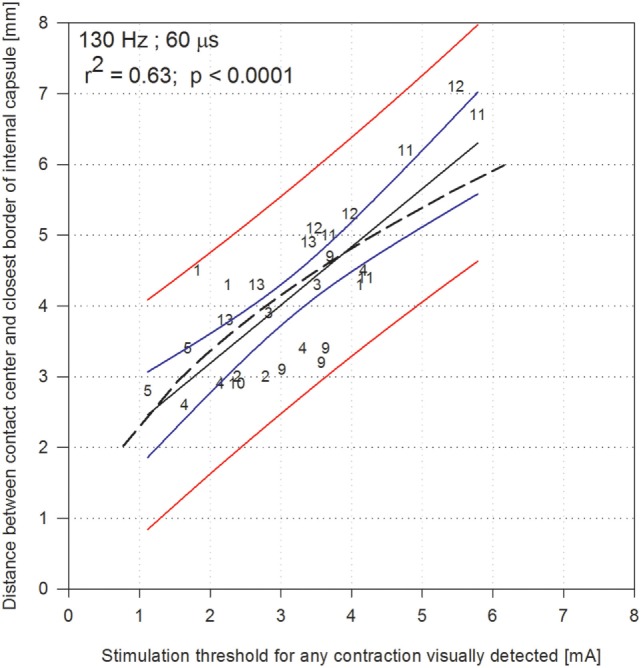
Shortest distance between the electrode contact and the corticospinal/corticobulbar tracts (internal capsule), according to the stimulation thresholds expressed in current [milliamperes (mA)] obtained for each contact at 130 Hz with 60 µs pulse duration, when contractions were visually detected. Numbers refer to each of the 12 patients (patient 7 excluded, numbers go from 1 to 13). The model of Kuncel et al. is indicated in dashed line; it was adapted for stimulation currents considering an average impedance of 1,300 Ω. The black line indicate the linear regression, the blue lines indicate 95% confidence band of the regression, and the red lines indicate the 95% prediction band.

*The correlation put in bold is the one represented in graph in Figure 3*.

The highest correlation coefficient was found for the visually detected contractions evoked at HFS when stimulation was described with estimated current (Pearson coefficient: 0.63, *p* < 0.0001; Spearman’s coefficient: 0.72, *p* < 0.001; Figure 3). Within Figure 3, the 95% prediction bands (red lines) can be used to determine from the stimulation threshold amplitude (milliamperes), the range within which the distance (millimeters) of the center of a contact to the closest border of the CSBT would be encountered. For comparison, we analyzed how the well accepted model of electrical activation described by Kuncel et al. (17) fitted with our data. The Pearson coefficient found was of *r*^2^ = 0.50 (Figure 3).

For all correlations with level of significance higher than 0.01, coefficients were found higher when stimulation was described with estimated current rather than with voltage (Table 1). This could be expected since, when using estimated currents, the variance due to variable impedance across patients is excluded, which is not the case with voltages.

Correlation coefficients obtained with LFS were not as high as with HFS (0.45 and 0.50 versus 0.54 with voltages; 0.43 and 0.61 versus 0.72 with currents; Table 1); however, the use of EMG when using LFS improved those correlations (Table 1). Indeed, with LFS-60, the visually detected contractions were seen far more often in the hand than the face (32 hand occurrences versus 5 face occurrences; *p* < 0.01; Table 2); once EMG detected, these proportions normalized (14 hand occurrences versus 10 face occurrences; *p* > 0.05), suggesting that EMG contributed to a better detection of contractions of the face at LFS. This was also the case with LFS-210 (33 hand occurrences versus 2 face occurrences; *p* < 0.01; Table 2); once EMG detected, these proportions normalized (19 hand occurrences versus 14 face occurrences; *p* > 0.05). These results approach the proportion seen with HFS, with which the contractions were slightly more frequently observed in the face than the hand (difference not significant; 19 face versus 10 hand occurrences; *p* > 0.05). As described in Table 2, in some patients, for given parameters, no contraction could be observed before other side-effects occurred (such as dizziness or ill-being sensation), explaining the reduced number of patients providing contraction thresholds.

**Table 2:** Distribution of the localization (eye, mouth, or hand) of the responses visually or electromyography (EMG) detected.

	Face (*N*) (%)		Hand (*N*) (%)	Overall (*N*)	Number of patients (max = 12)
	Eye	Mouth			
High-frequency stimulation visually detected	6	13	10	29	10
	**65.5**		**34.5**		
Low-frequency stimulation (LFS)-60 visually detected	4	1	32	37	11
	**13.5**		**86.5**		
LFS-60 EMG detected	1	9	14	24	7
	**41.7**		**58.3**		
LFS-210 visually detected	2	0	33	35	10
	**5.7**		**94.3**		
LFS-210 EMG detected	4	10	19	33	8
	**42.4**		**57.6**		

*The number of patients for whom contractions could be observed, either visually or with EMG, is indicated in every stimulation condition*.

*Percentages are provided in bold*.

With HFS, the contractions were, for the hand, tonic contractions inducing slow movements (e.g., abduction of the index finger in case of first dorsal interosseus contraction; opposition of the thumb in case of thenar group contraction); for the face they consisted of small twitches or “fibrillations” of small muscle parts, usually around the corner of the lip, just above or below it. With LFS, the induced contractions were myoclonic jerks of the hand muscles. At threshold, the jerks would be intermittent; if the amplitude was increased any further, then the myoclonic jerks would become permanent at the given 2 Hz frequency. For the hand, it was thus much easier to identify the contractions with LFS than with HFS: the former eliciting rhythmic jerks and the latter slow tonic contractions, whose threshold it was difficult to identify. For the face, on the contrary, the small twitches were easily seen with HFS.

## Discussion

The present study confirms a linear correlation between the stimulation amplitude and the distance of the DBS electrode to the CSBT. It provides a prediction band that could be used intraoperatively to ensure safe distance of the electrode to the CSBT, when the stimulation amplitudes were described with current (milliamperes) rather than voltage (volts). To the best of our knowledge, a correlation between the distance from the site of stimulation to the CSBT and stimulation amplitude has been studied in the field of DBS for movement disorders with amplitude of stimulation only reported as voltage (7). One modeling study of the spread of the electrical activation provides an equation linking the distance of one contact to the threshold of stimulation necessary to elicit paresthesia in thalamic stimulation (17). However, Kuncel et al. used 90 µs and 160 Hz to elicit paresthesia in thalamic stimulation (17), whereas here 60 µs and 130 Hz were used to elicit muscle contractions through CSBT stimulation. These differences in pulse duration and in stimulation frequency can explain partly the differences found with the equation described by Kuncel et al. (17). Also the comparison of real data with modeling studies remind us here that many other parameters can make reality different from what models can predict.

One similar linear correlation between the distance to the CSBT and the amplitude of stimulation has been studied in the field of intraoperative neuromonitoring for brain tumor neurosurgery with the purpose of preventing CSBT infarct (3–6). In this setting, CSBT mapping was performed for more distant stimulation, i.e., with larger amplitudes of stimulation and with different stimulation parameters (up to 25 mA, short trains of five pulses, rather than continuous stimulation). In our study, such a correlation was found with much smaller ranges of stimulation, which is of major interest to preserve the nearby CSBT (18).

Correlation coefficients were higher when stimulation was described with estimated current. The variance due to heterogeneous impedance across patients is excluded with currents but not with voltages. This illustrates the clinical implications of using voltage-controlled DBS rather than current-controlled DBS. Indeed, in voltage-controlled DBS, the amount of current that spreads throughout brain tissue is dependent upon the impedance of the tissue surrounding the electrode contact, whereas in current-controlled DBS, the IPG delivers a constant amount of current, irrespective of the impedance (19). Impedance varies with stimulation parameters, type of tissue, or time (19, 20). For instance, the scar surrounding a chronically implanted electrode may vary the impedance. With stimulation duration, impedance can change (21–23), in particular, when a contact starts to be stimulated (24, 25), or even during sleep (26). It is noteworthy that the stimulation thresholds could vary according to the alertness of our patients, possibly due to changes in impedance or muscle relaxation. To avoid that, we attempted to maintain patients’ alertness. In the present study, the stimulation amplitude was applied as voltage, due to technical considerations, and the thresholds expressed as current are the result of a calculation including the measurement of the impedances. An even more precise correlation might have been obtained with patients equipped with current-delivering IPGs.

The present study confirms that the clinical use of HFS to detect CSBT stimulation from visually detected contractions is a marker for localization of DBS electrodes. Nevertheless, the use of LFS with EMG-detected contractions can also be applied.

The shortest distance of each electrode contact to the CSBT was determined on an axial plane: indeed the coronal plane can also be reliably used for the ventral contacts as these contacts are closest to the medial border of the CSBT. However, for dorsal contacts, an axial plane should be used, principally since they are closest to the antero-medial border of the CSBT (Figure 2C); moreover, the medial border of the CSBT is less visible dorsally on a coronal plane. Tommasi et al. (7) used a coronal plane, and that technique was reliable for lower contacts, less so for more dorsal contacts. Therefore, we suggest using an axial plane.

### Limitations of the Study

First, clinical contractions were only searched on the face and on the contralateral hand; scrutinizing the foot or other body parts would have not been feasible in practice. Therefore, this study cannot account for all types of CSBT contractions. Second, there were outsiders (data not shown) that contributed to decrease the coefficient correlations at LFS; they were probably due to missed detection of face contractions (Table 2), contributing to assign higher currents to the measured electrode distance to the CSBT. Third, EMG detection of contractions was especially useful in the case of patients with mild to moderate tremor, which impaired visual detection of contractions (Figure 1); tremor interfering with visual detection could have also contributed to assign higher thresholds of contraction detection. Fourth, regarding the distance measurement on the fused CT-MRI, there is an inherent risk combining the CT and MRI images; however, this was indispensable.

## Conclusion

This study found a significant linear correlation between the distance of the DBS electrode contact to the CSBT and the stimulation amplitude. It provides a prediction band to ensure minimum distance of the DBS contacts to the CSBT from the stimulation amplitude. This correlation was stronger when stimulation was described with estimated current (milliamperes), reinforcing the need for current-controlled stimulation when conducting across patients or intraoperative DBS studies. The same data were close to a model of electrical spread of activation (17). We confirmed that the HFS chronically used for DBS can be applied to assess CSBT side-effects related to DBS, as is done intraoperatively, but should be considered respecting a prediction band and not simply an equation. Whereas this correlation was not superior with LFS compared to HFS, the use of surface EMG seems to contribute to more precise correlations at LFS than when contractions were visually detected, as EMG contributed to detect more often facial contractions.

As suggested by studies conducted in the field of tumoral neurosurgeries, such a prediction band could be applied during DBS procedures performed under general anesthesia as a tool to ensure safe distance of the electrodes to the CSBT.

## Ethics Statement

This study was conducted according to ethical guidelines of the Declaration of Helsinki and was approved by the Ethical Committee of the University Hospitals of Geneva (ref. no. 14-230). All patients gave and signed an informed consent.

## Author Contributions

JB: research project, organization, execution, design, review and critique, writing of the first draft; M-IV: organization, execution, review and critique; HJ, VF, PB, and SM: conception, review and critique; PP: conception, design, review and critique; CB: conception, organization, execution, design, review and critique.

## Conflict of Interest Statement

The authors declare that the research was conducted in the absence of any commercial or financial relationships that could be construed as a potential conflict of interest.

## References

[1] Pollak P Krack P Fraix V Mendes A Moro E Chabardes S Intraoperative micro. Mov Disord (2002) 17(S3):S155–61. 10.1002/mds.10158 11948771

[2] Mehanna R Machado AG Connett JE Alsaloum F Cooper SE Intraoperative microstimulation predicts outcome of postoperative macrostimulation in subthalamic nucleus deep brain stimulation for Parkinson’s disease. Neuromodulation (2017). 10.1111/ner.12553 28093818

[3] Seidel K Beck J Stieglitz L Schucht P Raabe A The warning-sign hierarchy between quantitative subcortical motor mapping and continuous motor evoked potential monitoring during resection of supratentorial brain tumors: clinical article. J Neurosurg (2013) 118(2):287–96. 10.3171/2012.10.JNS12895 23198802

[4] Kamada K Todo T Ota T Ino K Masutani Y Aoki S The motor-evoked potential threshold evaluated by tractography and electrical stimulation: clinical article. J Neurosurg (2009) 111(4):785–95. 10.3171/2008.9.JNS08414 19199462

[5] Nossek E Korn A Shahar T Kanner AA Yaffe H Marcovici D Intraoperative mapping and monitoring of the corticospinal tracts with neurophysiological assessment and 3-dimensional ultrasonography-based navigation: clinical article. J Neurosurg (2011) 114(3):738–46. 10.3171/2010.8.JNS10639 20799862

[6] Ohue S Kohno S Inoue A Yamashita D Harada H Kumon Y Accuracy of diffusion tensor magnetic resonance imaging-based tractography for surgery of gliomas near the pyramidal tract: a significant correlation between subcortical electrical stimulation and postoperative tractography. Neurosurgery (2012) 70(2):283–94. 10.1227/NEU.0b013e31823020e6 21811189

[7] Tommasi G Krack P Fraix V Le Bas JF Chabardes S Benabid AL Pyramidal tract side effects induced by deep brain stimulation of the subthalamic nucleus. J Neurol Neurosurg Psychiatry (2008) 79(7):813–9. 10.1136/jnnp.2007.117507 17928327

[8] Odekerken VJ van Laar T Staal MJ Mosch A Hoffmann CF Nijssen PC Subthalamic nucleus versus globus pallidus bilateral deep brain stimulation for advanced Parkinson’s disease (NSTAPS study): a randomised controlled trial. Lancet Neurol (2013) 12(1):37–44. 10.1016/S1474-4422(12)70264-8 23168021

[9] Krack P Batir A Van Blercom N Chabardes S Fraix V Ardouin C Five-year follow-up of bilateral stimulation of the subthalamic nucleus in advanced Parkinson’s disease. N Engl J Med (2003) 349(20):1925–34. 10.1056/NEJMoa035275 14614167

[10] Deep-Brain Stimulation for Parkinson’s Disease Study Group. Deep-brain stimulation of the subthalamic nucleus or the pars interna of the globus pallidus in Parkinson’s disease. N Engl J Med (2001) 2001(345):956–63. 10.1056/NEJMoa000827 11575287

[11] Kleiner-Fisman G Herzog J Fisman DN Tamma F Lyons KE Pahwa R Subthalamic nucleus deep brain stimulation: summary and meta-analysis of outcomes. Mov Disord (2006) 21(S14):S290–304. 10.1002/mds.20962 16892449

[12] Khan FR Henderson JM Deep brain stimulation surgical techniques. In: Lozano AM Hallett M, editors. Handbook of Clinical Neurology. Brain Stimulation. (Vol. 116) Amsterdam: Elsevier (2013). p. 27–37. 10.1016/B978-0-444-53497-2.00003-6 24112882

[13] Benabid AL Krack PP Benazzouz A Limousin P Koudsie A Pollak P Deep brain stimulation of the subthalamic nucleus for Parkinson’s disease: methodologic aspects and clinical criteria. Neurology (1999) 55(12 Suppl 6):S40–4. 11188974

[14] Butson CR McIntyre CC Differences among implanted pulse generator waveforms cause variations in the neural response to deep brain stimulation. Neurophysiol Clin (2007) 118(8):1889–94. 10.1016/j.clinph.2007.05.061 PMC2075350 17581776

[15] Tyrand R Seeck M Spinelli L Pralong E Vulliemoz S Foletti G Effects of amygdala–hippocampal stimulation on interictal epileptic discharges. Epilepsy Res (2012) 99(1):87–93. 10.1016/j.eplepsyres.2011.10.026 22079883

[16] Barnaure I Pollak P Momjian S Horvath J Lovblad KO Boëx C Evaluation of electrode position in deep brain stimulation by image fusion (MRI and CT). Neuroradiology (2015) 57(9):903–8. 10.1007/s00234-015-1547-z 26022355

[17] Kuncel AM Cooper SE Grill WM A method to estimate the spatial extent of activation in thalamic deep brain stimulation. Neurophysiol Clin (2008) 119(9):2148–58. 10.1016/j.clinph.2008.02.025 PMC2587000 18632304

[18] Raabe A Beck J Schucht P Seidel K Continuous dynamic mapping of the corticospinal tract during surgery of motor eloquent brain tumors: evaluation of a new method: clinical article. J Neurosurg (2014) 120(5):1015–24. 10.3171/2014.1.JNS13909 24628613

[19] Brocker DT Grill WM Principles of electrical stimulation of neural tissue. In: Lozano AM Hallett M, editors. Handbook of Clinical Neurology. Brain Stimulation. (Vol. 116) Amsterdam: Elsevier (2013). p. 3–18. 10.1016/B978-0-444-53497-2.00001-2 24112880

[20] Lempka SF Miocinovic S Johnson MD Vitek JL McIntyre CC. In vivo impedance spectroscopy of deep brain stimulation electrodes. J Neural Eng (2009) 6(4):046001. 10.1088/1741-2560/6/4/046001 19494421 PMC2861504

[21] Cheung T Nuño M Hoffman M Katz M Kilbane C Alterman R Longitudinal impedance variability in patients with chronically implanted DBS devices. Brain Stimulat (2013) 6(5):746–51. 10.1016/j.brs.2013.03.010 23619246

[22] Satzer D Lanctin D Eberly LE Abosch A. Variation in deep brain stimulation electrode impedance over years following electrode implantation. Stereotact Funct Neurosurg (2014) 92(2):94–102. 10.1159/000358014 24503709 PMC4531050

[23] Satzer D Maurer EW Lanctin D Guan W Abosch A Anatomic correlates of deep brain stimulation electrode impedance. J Neurol Neurosurg Psychiatry (2014) 86(4):398–403. 10.1136/jnnp-2013-307284 24935985 PMC13349391

[24] Jaggi JL Baltuch GH Deep brain stimulation inactivity can produce unexpected high electrode impedances when reactivated, leading to a false conclusion of wire fracture. Stereotact Funct Neurosurg (2006) 83(5–6):187–9. 10.1159/000090433 16374074

[25] de Sauvage RC da Costa DL Erre JP Aran JM. Changes in CM and CAP with sedation and temperature in the guinea pig: facts and interpretation. Hear Res (1996) 102(1):15–27. 10.1016/S0378-5955(96)00137-2 8951446

[26] Ranck JB Electrical impedance changes in many sites of brain in paradoxical sleep, anesthesia, and activity. Exp Neurol (1970) 27(3):454–75. 10.1016/0014-4886(70)90107-X 4316736

